# Supplementary material for: Comparison of hematological parameters between type 2 diabetes mellitus patients and healthy controls at Dessie comprehensive specialized hospital, Northeast Ethiopia: Comparative cross-sectional study
Source: PLoS One. 2022 Jul 27;17(7):e0272145. doi: 10.1371/journal.pone.0272145 (PMC9328522; doi:10.1371/journal.pone.0272145)
Supplement: S1 File — (DOCX) [file pone.0272145.s001.docx]

## Questionnaires

**Part I:** This questionnaire is prepared to carry out a study to compare hematological parameters between type 2 diabetes mellitus patients and healthy control at Dessie comprehensive specialized hospital, Northeast Ethiopia

Identification code: _______________

**Table 1: Socio-demographic characteristics of study participant**

| S.no | **Variable**s | **Possible responses** |
| --- | --- | --- |
|  | Age | ------------ |
|  | Sex | 1. Male 2. Female |
|  | Residence | 1. Urban 2. Rural |
|  | Educational status | 1. Not read and write 2. Primary school 3. Secondary school 4. Diploma and above |
|  | Occupational status | 1. Student 2. Non-employed worker 3. Employed worker |

**Part II:** Checklist prepared to collect anthropometric and clinical variables from the medical record of type 2 diabetic patients at Dessie comprehensive specialized hospital, northeast Ethiopia.

Identification code_______________

**Table 2: Clinical and anthropometric variables of T2DM study participant**

| **S.no** | **Variable**s | **Possible Responses** |
| --- | --- | --- |
|  | Diabetic ketoacidosis | 1. Yes 2. No |
|  | Foot ulcer | 1. Yes 2. No |
|  | Hyperosmaolar hyperglycemic state (HHS) | 1. Yes 2. No |
|  | Neuropathy | 1. Yes 2. No |
|  | Visual disturbance | 1. Yes 2. No |
|  | Systolic blood pressure (mmHg) | 1. ------ |
|  | Diastolic blood pressure (mmHg) | 1. ------ |
|  | Body mass index (BMI (Kg/m^2^)) | 1. ------- |
|  | Treatment regimen | 1. Insulin 2. Oral hypoglycemic agents 3. Mixed regimen (insulin and oral agents) |
|  | Duration of the disease (years) | 1. ------- |

**Part III:** Checklist prepared to collect hematological and fasting blood glucose level of type 2 diabetes patients and controls at Dessie comprehensive specialized hospital, northeast Ethiopia.

Identification code_______________

**Table 3: Hematological and fasting blood glucose level of study participants**

| S.no | **Parameters** | **Results** |
| --- | --- | --- |
|  | Total WBC count (10^9^/L) | 1. --------- |
|  | Neutrophil count (10^9^/L) | 1. --------- |
|  | Lymphocyte count (10^9^/L) | 1. --------- |
|  | Monocye count (10^9^/L) | 1. --------- |
|  | Eosinophil count (10^9^/L) | 1. --------- |
|  | Basophil count (10^9^/L) | 1. --------- |
|  | Neutrophil % | 1. --------- |
|  | Lymphocyte % | 1. --------- |
|  | Monocyte % | 1. --------- |
|  | Eosinophil % | 1. --------- |
|  | Basophil % | 1. --------- |
|  | RBC count (10^12^/L) | 1. --------- |
|  | Hemoglobin level (g/dL)) | 1. --------- |
|  | Hematocrit level (%) | 1. --------- |
|  | MCV (fL) | 1. --------- |
|  | MCH (Pg) | 1. --------- |
|  | MCHC (g/dL) | 1. --------- |
|  | Platelet count (10^9^/L) | 1. --------- |
|  | RDW-SD (fL) | 1. --------- |
|  | RDW-CV (%) | 1. --------- |
|  | PDW (fL) | 1. --------- |
|  | MPV(fL) | 1. --------- |
|  | PLC-R (%) | 1. --------- |
|  | Plateletcrit (%) | 1. --------- |
|  | FBG (mg/dL) | 1. --------- |
