# Supplementary material for: Comparison of hematological parameters between type 2 diabetes mellitus patients and healthy controls at Dessie comprehensive specialized hospital, Northeast Ethiopia: Comparative cross-sectional study
Source: PLoS One. 2022 Jul 27;17(7):e0272145. doi: 10.1371/journal.pone.0272145 (PMC9328522; doi:10.1371/journal.pone.0272145)
Supplement: Attachment — Submitted filename: Response to Reviewers.docx [file pone.0272145.s004.docx]

**Point by point response to reviewers and editors**

**Dear Editor and reviewers:**

First, we would like to give our deepest gratitude to the reviewers and editors for their time and dedication to providing valuable and constructive comments. We authors addressed all the concerns raised by **editors** and **reviewers** point-by-point as follows.

**Response to Reviewers**

**Reviewer #1**

**Comment #1:** I consider that the work done by Hussen and coworkers is important because they are trying to exploit data acquired from the hematological test that are a useful available test, but the document needs to be improved. It seems that the main objective of the study was set after the authors concluded the statistical analysis, this comment is based on the lack of sufficient information regarding anemia in the population studied. In the introduction, the authors also indicate that there are commonly two types of diabetes, according to ADA there are 4 general categories, see American Diabetes Association Professional Practice Committee; 2. Classification and Diagnosis of Diabetes: Standards of Medical Care in Diabetes—2022. Diabetes Care 1 January 2022; 45 (Supplement_1): S17–S38. https://doi.org/10.2337/dc22-S002. The authors also said that T1DM affects commonly children and adolescents and T2DM affects young adults. This statement is no longer accurate, as both diseases occur in both age groups (see the reference above). I consider that reference 4 does not support the information about the diabetes classification. There is also a 2021 version of the IDF atlas; I think it is a good idea to actualize the reference information.

**Response #1:** Thank you for your valuable and constructive comments. Yes, we authors tried to revise and make amendments based on the main concern raised by the reviewer. First, we would like to say thank you to the reviewer for constructive comments which could help us to improve the overall quality of our paper. Thus, in the first paragraph of the introduction part, the authors used the traditional diabetes classification scheme and definition of diabetes It was the two types of diabetes; T1DM and T2DM that are considered to be the main diabetes diagnosed across the world before but now, we have changed the diabetes classification scheme and their definitions according to the current IDF atlas,2021 and ADA professional practice, 2022 diabetes classification and definition recommendations that were recommended by the reviewer to use these guidelines. Therefore, the changes have been included in the revised manuscript.

**Comment #2:** Study participants: The authors did not make an appropriate description of the clinical characteristics of the population studied. They should include the inclusion criteria. Moreover, they need to include the values of FBG, HbA1c and explain how they evaluated the existence of diabetic complications it is hard to believe that the patients presented neuropathy, hyperosmolar hyperglycemia, foot ulcers, etc. and they do not present a certain degree of renal dysfunction; it is well known that renal dysfunction is a cause of anemia. Few patients reported those kinds of complications, if the authors exclude those patients, will they get the same results?

**Response #2:** Thank you for your valuable and constructive comments. Yes, the method part describing the study participants believed to be not complete specifically does not provide comprehensive information regarding the source population for diabetic and control groups as well as the inclusion criteria for including study participants in both diabetic and control groups. To highlight, the source population for this study was all T2DM patients visiting the hospital for follow-up service included in the diabetic group, and all healthy blood donors visiting Dessie blood bank for blood donation included in the control group. The study was conducted on a total of 240 study participants consisting of 120 T2DM attending for follow up in diabetes clinics of the hospital and 120 healthy controls attending at Dessie blood bank for blood donation who fulfilled the eligibility criteria were included in the study. The FBG value for both T2DM and the healthy group was described in the manuscript result section of Table 3 as the mean and standard deviation for T2DM is 264.0±(94.90) and for healthy control is 87.97 ± (8.45) but the value of HbA1c was not mentioned. Because our country is included in developing countries, HbA1c is not done as part of routine laboratory diagnostic tests due to cost affordability and accessibility problems. As an alternative, FBG is routinely done to diagnose and monitor diabetes-related complications. Since the authors collected the clinical related variables (diabetic complications) of T2DM from patient clinical information sheet (secondary data) which was already described and characterized by their respective physicians or clinicians as the clinician utilized the diabetes guideline to assess the diabetic associated complications. Yes, it is a good suggestion that nephropathy is the main cause of not only anemia but also affects the platelet count, platelet indices, RBC parameters, and leucocyte parameters. Therefore, the authors are interested to assess the hematological alterations in T2DM patients without nephropathy but, the authors took your recommendation and tried to investigate the hematological alterations in T2DM patients by including those of diabetes with nephropathy in the future study. For now, the authors take this point as a limitation of the study.

**Comment #3:** Sample size: The authors should include the statistical formulae used to estimate the sample size and which variables they took into account to perform the estimation of the sample size and support the information with reference values. Concerning the statistical analysis, I do not have the expertise to consider them.

**Response #3:** Thank you for your constructive comments and suggestions. Our study is a comparative cross-sectional containing two groups that include T2DM patients and healthy controls. The authors tried to calculate sample size by using the double population proportion formula and or double population mean formula. Unfortunately, using the two formulas to calculate the sample size gives a small sample size for our study. Thus, the authors find out an alternative option to solve this problem and they used rule of thumb assumptions drawn by van Voorhis and Morgan has been applied to determine the sample size. According to the rule, at least 30 study participants per group are required to detect real differences which could lead to about 80% power if the study contains two or more groups and if they used T-test, ANOVA…etc as statistical tools and therefore the authors increased the sample size by fourfold in each study group recommended by van Voorhis and Morgan to increase the reliability of the study. This description is mentioned in the method part of the sample size determination already cited.

**Comment #4:** Figures and tables: The authors need to detail the format presentation of the variables, Table 1 is not well introduced in the text; furthermore, line one of this table lacks a variable title, what does < 45 mean? I suggest including the age range. Authors should uniform the title of the variables in the tables, sometimes they write with uppercase and in other places of the document with lowercase. There is a format error in table 2.

**Response #4:** Thank you for your valuable comments. Based on the comment, the authors carried out a thorough revision and made changes regarding inconsistency such as missing the age in under variable in Table 1 as well as inconsistency in writing using uppercase and the lower case has been revised throughout the document and the changes are included in the revised manuscript.

**Comment #5:** In the discussion section, they did not argue about the limitations of the study nor the strengths, and in the conclusion section, the authors seem to summarize the results. References should be written according to the requirements of the journal.

**Response #5:** Thank you for your valuable suggestions and comments. The authors describe and include the limitations as well as the strengths of the study at the end of the discussion. The conclusion section has been revised according to the finding of the study. References are written according to PLOS ONE journal referencing style and requirements
