# Supplementary material for: Comparison of hematological parameters between type 2 diabetes mellitus patients and healthy controls at Dessie comprehensive specialized hospital, Northeast Ethiopia: Comparative cross-sectional study
Source: PLoS One. 2022 Jul 27;17(7):e0272145. doi: 10.1371/journal.pone.0272145 (PMC9328522; doi:10.1371/journal.pone.0272145)
Supplement: Attachment — Submitted filename: Response to Reviewers - R2.docx [file pone.0272145.s005.docx]

**A point-by-point response to reviewers and editors**

**Dear Editor and reviewers:**

First, we would like to give our deepest gratitude to the reviewers and editors for their time and dedication to providing valuable and constructive comments. The authors addressed all the concerns raised by **editors** and **reviewers** point-by-point as follows and the changes are indicated by red color in the revised manuscript.

**Response to Reviewers**

**Reviewer #1**

**Comment #1:** The authors improve the manuscript significantly, but there are some things to attend to. Introduction:- Even if the authors did not reply to my questions they took into account my suggestions actualizing the references about the classification of diabetes and the reference.

**Response #1:** Thank you for your valuable and constructive comments. Previously, the authors tried to elaborate queries regarding the classification systems of diabetes. Previous classification systems mainly classified diabetes into two main categories based on the level of insulin and the function of insulin (insulin resistance). For example, WHO published its first widely accepted and globally adopted classification of diabetes in 1980 and an updated version of this in 1985. These classifications included two major classes of diabetes: insulin-dependent diabetes mellitus (IDDM), or T1DM which mainly affects children, and non-insulin-dependent diabetes mellitus (NIDDM), or T2DM which commonly affects adolescents and old age individuals. Later, WHO and other diabetes guidelines revised the classification of diabetes by including other classes of diabetes categorized as “other types” and “gestational diabetes mellitus” (GDM). Previously, scholars may consider the age of the patient to classify diabetes as T1DM which may affect juveniles and T2DM which may affect adolescents and old ages. Currently, age could not be the basis for classification purposes due to both T1DM and T2DM affects any age group population. Generally, many diabetes guidelines recommended the classification of diabetes relies on three primary aims: (1) to guide clinical care decisions, (2) to stimulate research on pathophysiology, and (3) to provide a basis for epidemiological studies. Any classification system should be able to help with all three of these key activities, but at present, there are so many gaps in understanding the causes of diabetes. Based on your interesting recommendation, the authors have thoroughly revised the diabetes classification systems, and the changes are included in the revised manuscript which was properly cited by the suggested references.

**Comment #2:** Study population. - The population description was improved but there is a lack of some important conditions (my humble point of view), such as glycated hemoglobin to state the glycemic control of the population. This is important because of the macro and microvascular complications.

**Response #2:** Thank you for your valuable comments. The point that was raised by the reviewer is acceptable. Accordingly, WHO and IDF are currently recommended four laboratory diagnostic tests for diabetes including measurement of fasting plasma glucose; 2-hour post-load plasma glucose after a 75-gram oral glucose tolerance test (OGTT); hemoglobin A1c (HbA1c); and random blood glucose in the presence of signs and symptoms of diabetes. People with fasting plasma glucose values of ≥ 7.0 mmol/L (126 mg/dl), 2-hour post-load plasma glucose ≥ 11.1 mmol/L (200 mg/dl), HbA1c ≥ 6.5% (48 mmol/mol); or a random blood glucose ≥ 11.1 mmol/L (200 mg/ dl) in the presence of signs and symptoms are considered to have diabetes.

Glycated hemoglobin or hemoglobin A1C indicates the plasma glucose level of the previous 2 or 3 months of the patient. It also has a significant role in the diagnosis of diabetes, confirmation of prediabetes, and monitoring the glycemic level during usual glucose-lowering treatment. It may also have a significant role in assessing the development of micro and macrovascular complications. In our study, glycated hemoglobin was not routinely performed determined due to constraints associated with the availability, cost affordability, availability of problem of laboratory equipment, and accessibility of the test in our country. Fasting blood glucose is routinely determined for diagnosis and monitoring of the glycemic level of the patient. Therefore, this could be an acceptable comment, and the authors considered it a limitation of the study.

**Comment #3:** Sample size.- The authors did not justify your strategy of “rule of the thumb”
Concerning the statistical analysis, I do not have the expertise to consider them.

**Response #3:** Thank you for your constructive comments and suggestions. When the researcher wanted to conduct the quantitative type of research, in the first place they should determine the number of study participants to be included in the study. To determine the sample size of the study, the researcher should consider different parameters such as generalizability, validity, reliability, power of the study, effect size, schedule, availability of resources, type of study design employed, the sample size, etc. Therefore, the researcher determined the sample size if the source population is too large that couldn’t be entirely included. Based on considering the above parameters, the authors used statistical formula i.e double population proportion or the double population mean formula to calculate the sample size by considering 95% CI, 5% of margin of error, and taking the significant hematological parameters as significant variables from previous studies. Unfortunately, using the above formula and taking the different hematological parameters as significant variables from different studies, we have got a small sample size to make inferences. So, the authors used a rule of thumb as an alternative means to determine the sample size.

The rules of thumb outline the key relationships between the determinants of statistical power and sample size and demonstrate how to design a high-powered randomized evaluation. According to "J-PAL. 2017. “Sampling and Sample Size.” Accessed May 26. https:// [www.povertyactionlab.org/sites/default/files/documents/L5_Sampling%20and%20Sample_Glennerster](http://www.povertyactionlab.org/sites/default/files/documents/L5_Sampling%20and%20Sample_Glennerster) 2016-06-15.pdf” six rules of thumb were considered before determining sample size and statistical power. Rule of Thumb #1: A larger sample increases the statistical power of the evaluation. Rule of Thumb #2: If the effect size of a program is small, the evaluation needs a larger sample to achieve a given level of power. Rule of Thumb #3: An evaluation of a program with low take-up needs a larger sample. Rule of Thumb #4: If the underlying population has a high variation in outcomes, the evaluation needs a larger sample. Rule of Thumb #5: For a given sample size, power is maximized when the sample is equally split between the treatment and control groups. Rule of Thumb #6: For a given sample size, randomizing at the cluster level as opposed to the individual level reduces the power of the evaluation.

The relationship between the sample size and rules of thumb depends on the study design used, statistical tools to be used, number of groups, etc…. If the research is focused on measuring the group differences, the sample size of 30 per group is required to make 80% power of the study (statistical tools e.g., t‐test, ANOVA), and if the research is focused on determining the relationship or correlation, the minimum sample size should be 50 to make 80% power of the study. According to the rule, at least 30 study participants per group are required to detect real differences which could lead to about 80% power if the study contains two or more groups and if they used T-test, ANOVA…etc as statistical tools and therefore the authors increased the sample size by fourfold making 120 sample size in each study group recommended by van Voorhis and Morgan to increase the reliability of the study. This description is mentioned in the method part of the sample size determination already cited.

**Comment #4:** Figures and tables.- In table 1, the authors changed the number of subjects that were under 45 years old. In the first version, the number was inverted. How do they explain this?
**Response #4:** Thank you for your valuable comments. During the first write-up of the manuscript, the age classification might face errors that happened unintentionally as a typing error. During the revision process, the authors found that the age classification was inverted inappropriately due to typing errors. Therefore, the authors fixed the error and corrected it by inverting the age category that was indicated by the highlight color while resubmitting the revised version of the manuscript.

**Comment #5:** Discussion.- The authors stated that poorly controlled patients are at higher risk of complications such as endothelial dysfunction and an increase in oxidative stress, but they omitted to describe the control degree of the population included in this study.

**Response #5:** The present study aims to assess the hematological parameters among T2DM patients and compare their hematological parameters with controls. This study did not assess the endothelial dysfunction and the level of oxidative stress. But, various studies indicated that poor glycemic control had a direct impact on blood vessels, and change the level of oxidative stress that may be directly associated with the increased risk of various complications compared to the controls concerning vascular, cellular, immunological, and other biochemical associated complications

**Comment #6:** In table 4 the Hgb did not correlate negatively with BMI the correlation was positive (r= 0.284) see table 4. The study of Adane T, Getawa S. Anaemia, and its associated factors among diabetes mellitus patients in Ethiopia: A systematic review and meta-analysis. Endocrinol Diabetes Metab. 2021 May 14;4(3):e00260. doi: 10.1002/edm2.260. found an odds for anemia of 2.98 and a major risk in males >60 years old and who had an illness duration of more than five years. This study found that anemia was more frequent in females than in males and at a younger age. Might the authors explain why? I think the contrasting results are important. In this sense, the conclusion would emphasize the importance of the diabetes follow-up in the early stages of the illness and why this study is relevant, I feel this is still the same as the past version but without numbers.

**Response #6:** Thank you for your valuable comments. We have assessed back the concern raised in table 4 regarding the correlation between hemoglobin and BMI. Yes, hemoglobin is positively correlated with BMI indicated by the correlation coefficient r=0.284. Even though they are positively correlated, they have a weak correlation shown by r=0.284. Various evidence showed that the degree of correlation is categorized using correlation coefficient: High degree if the coefficient value lies between ± 0.50 and ± 1, then it is said to be a strong correlation, moderate degree if the value lies between ± 0.30 and ± 0.49, then it is said to be a medium correlation and low degree when the value lies below + 0. 29, then it is said to be a small correlation.

Yes, a systematic review and meta-analysis did by Adane T et al, about Anaemia, and its associated factors among diabetes mellitus patients in Ethiopia indicated that the prevalence of anemia was higher among males (36.72% [95% CI: 22.58–50.87] I2 = 97.6%) than female (27.51% [95% CI: 16.12– 38.90] I2 = 96.3%). Moreover, the odds of anaemia were higher among patients with age ˃ 60 (OR = 2.98; 95% CI: 1.83, 4.87), and duration of illness ≥5 years (OR = 2.66; 95%: 1.38, 5.13). In contrast, another systematic review and meta-analysis done by Atlaw et al about the magnitude and factors associated with anemia among diabetic patients in Ethiopia: A systematic review and meta-analysis. *SAGE Open Medicine*. January 2021. doi:[10.1177/20503121211031126](https://doi.org/10.1177/20503121211031126). The meta-analysis has revealed that there was no difference among male and female diabetic patients in the occurrence of anemia (POR, 95% CI: 1.2 (0.94–1.52)) with a significant level of heterogeneity (*I*^2^ = 89%, *p* < 0.001), age greater than 60 years
old (pooled odds ratio, 95% confidence interval: 3.73 (2.23–6.77)), glomerular filtration rate less than 60 mL/min/1.73 m2 (pooled odds ratio, 95% confidence interval: 12.65 (8.71–18.37)), and being diabetic for more than 10 years (pooled odds ratio, 95% confidence interval: 10.21 (7.00–15.04)) were found to be determinants of anemia among diabetic patients in Ethiopia. In the current finding, anemia was found higher in female than male study participants but, there was no statistically difference between male and female patients that was indicated by AOR=0.35, (95% CI=0.10-2.14, P=0.25), and anemia was found to be higher with age ≤45 compared to the age >45 but there was no statistical difference which was indicated by AOR =2.7 (95% CI=0.20-37.5, P=0.46). The possible variation might be the current study is a single hospital-based cross-sectional study while Adane T, Getawa S was done a systematic review and meta-analysis that showed the pooled prevalence. moreover, other possible factors may contribute to the variations such as variation in the sample size, study design, laboratory diagnostic method used, etc….

**Comment #7:** There are some errors in the references please check the style and numeration.

**Response #7:** We have checked the references and we found some errors. Therefore, we revised thoroughly the references accordingly to the journal references requirements and styles.
